# Simultaneous heart-kidney transplantation results in respectable long-term outcome but a high rate of early kidney graft loss in high-risk recipients – a European single center analysis

**DOI:** 10.1186/s12882-021-02430-x

**Published:** 2021-07-09

**Authors:** Oliver Beetz, Juliane Thies, Clara A. Weigle, Fabio Ius, Michael Winkler, Christoph Bara, Nicolas Richter, Jürgen Klempnauer, Gregor Warnecke, Axel Haverich, Murat Avsar, Gerrit Grannas

**Affiliations:** 1 Department of General, Visceral and Transplant Surgery, Hannover Medical School, Carl-Neuberg-Str. 1, 30626 Hannover, Germany; 2 Department of Cardiothoracic, Transplant and Vascular Surgery, Hannover Medical School, Hannover, Germany; 3 Department of Cardiac Surgery, University of Heidelberg, Heidelberg, Germany

**Keywords:** Multivisceral transplantation, Kidney transplantation, Heart transplantation

## Abstract

**Background:**

In spite of renal graft shortage and increasing waiting times for transplant candidates, simultaneous heart and kidney transplantation (HKTx) is an increasingly performed procedure established for patients with combined end-stage cardiac and renal failure. Although data on renal graft outcome in this setting is limited, reports on reduced graft survival in comparison to solitary kidney transplantation (KTx) have led to an ongoing discussion of adequate organ utilization.

**Methods:**

This retrospective study was conducted to evaluate prognostic factors and outcomes of 27 patients undergoing HKTx in comparison to a matched cohort of 27 patients undergoing solitary KTx between September 1987 and October 2019 in one of Europe’s largest transplant centers.

**Results:**

Median follow-up was 100.33 (0.46–362.09) months. Despite lower five-year kidney graft survival (62.6% versus 92.1%; 111.73 versus 183.08 months; *p* = 0.189), graft function and patient survival (138.90 versus 192.71 months; *p* = 0.128) were not significantly inferior after HKTx in general. However, in case of prior cardiac surgery requiring sternotomy we observed significantly reduced early graft and patient survival (57.00 and 94.09 months, respectively) when compared to patients undergoing solitary KTx (183.08 and 192.71 months; *p* < 0.001, respectively) or HKTx without prior cardiac surgery (203.22 and 203.22 months; *p* = 0.016 and *p* = 0.019, respectively), most probably explained by the significantly increased rate of primary nonfunction (33.3%) and in-hospital mortality (25.0%).

**Conclusions:**

Our data demonstrates the increased rate of early kidney graft loss and thus significantly inferior graft survival in high-risk patients undergoing HKTx. Thus, we advocate for a “kidney-after-heart” program in such patients to ensure responsible and reasonable utilization of scarce resources in times of ongoing organ shortage crisis.

**Supplementary Information:**

The online version contains supplementary material available at 10.1186/s12882-021-02430-x.

## Background

End-stage renal insufficiency in patients undergoing heart transplantation (HTx) is accompanied by poor postoperative survival of approximately 50% after 12 months [1, 2]. Accordingly, simultaneous heart-kidney transplantation (HKTx) was introduced to improve patient outcome. Data obtained from the Organ Procurement and Transplantation Network revealed a nearly fivefold increase of HKTx from 2004 (44) to 2018 (202), equivalent to the most significant increase in all multi-organ transplants performed over the past years [3]. However, in comparison to solitary kidney-transplants (KTx) with low in-hospital mortality (0.8 to 1.4%) and satisfying 1-year patient survival of 96% [4], HKTx is accompanied by a severely increased in-hospital mortality of up to 22% [5] and thus, reduced 1-year survival rates ranging from 62 to 84% [1, 5–7]. In times of ongoing organ shortage, this data is alarming as the allocation of two organs to a single patient has to be justified not only with regard to patients awaiting HTx, but also to patients on the waiting list for KTx: Since kidney injury is frequently associated with end-stage heart failure and a growing number of patients experience prolonged waiting times for an appropriate cardiac transplant [8], concomitant demand of renal grafts for these patients is predictable, which will further diminish the availability of organs in the KTx allocation system [9]. This implicates not only socioeconomic ramifications and impairment of quality of life of potential recipients, but also significantly decreases long-term survival of patients requiring renal replacement therapy [10–12].

In 2009 Russo et al. analyzed the United Network for Organ Sharing database for prognostic factors impeding survival in patients undergoing HKTx and identified peripheral vascular disease, recipient age older than 65 years, non-ischemic etiology of heart failure, dialysis dependence at the time of transplantation, and bridge to transplantation using a ventricular assist device as poor prognostic factors with a 1-year-survival of only 61.9% questioning the legitimacy of simultaneous HKTx in such patients [13].

This is also accentuated by technical and surgical developments as well as prolonged waiting times prior to transplant leading to an increase of patients with further risk factors, such as previous operations and/or mechanical circulatory support before undergoing transplantation.

Although HKTx is performed more and more frequently, data on the matter is scarce and based mainly on national registries. Aim of this study was therefore to evaluate prognostic factors and outcomes of patients undergoing HKTx in comparison to patients undergoing solitary KTx in one of the largest transplant centers in Europe.

## Methods

### Recipients

We retrospectively examined data from patients who underwent HKTx and from a matched cohort of patients undergoing solitary KTx at the Hannover Medical School in the time period between September 1987 and October 2019. Matching was performed with respect to the date of transplant (+ 4 years), gender, age, body mass index (BMI), cold ischemic time, human leukocyte antigen (HLA)-mismatches, current and highest pre-existent percent panel reactive antibodies (%PRA) and donor time in intensive care unit (ICU). Cardiovascular risk factors assessed for each patient were smoking, diabetes, hypertension, hyperlipidemia and BMI > 30.

The peri- and postoperative course of each patient was analyzed based on electronic files from participating departments (Department of Cardiothoracic, Transplant and Vascular Surgery, Department of General, Visceral and Transplant Surgery, and interdisciplinary outpatient transplant clinic). HKTx was performed in case of end-stage cardiac failure and creatinine clearance of less than 40 ml/min, without expected renal improvement upon solitary HTx after evaluation at the Department of Nephrology prior to listing at Eurotransplant. In accordance with current allocation rules, all patients undergoing solitary KTx were on dialysis prior and at the time of transplant.

### Transplantation procedure

Patients undergoing HKTx received their renal graft from the identical donor either immediately or staged following a few hours break, depending on the duration of the HTx, quantity of intraoperatively transfused blood products as well as patient’s body temperature and hemodynamic situation.

### Postoperative follow-up

All patients included in the study were seen on regular basis in the interdisciplinary outpatient transplant clinic at the Hannover Medical School. Follow-up of patients included regular clinical investigation, routine laboratory tests and sonography. The estimated glomerular filtration rate (eGFR) was calculated by routinely measured creatinine levels. After HKTx regular echocardiography was carried out and endomyocardial biopsies were performed according to a standard surveillance protocol or upon suspected acute cardiac rejection. Cardiac rejection episodes were treated if greater than 1B, according to the classification of the International Society for Heart and Lung Transplantation. Renal allograft rejection was diagnosed by biopsy and treated accordingly. Follow-up time is reported in months and was defined as time between date of transplantation and date of last contact, graft loss/failure or death, respectively. Kidney graft survival was defined equivalent to graft loss and was thus not censored for patient death.

### Statistics

Upon collection of data, statistical analyses were performed with SPSS statistical software (version 26; SPSS Inc.; IBM Corporation, Armonk, NY USA). For comparison of categorical variables between groups chi-squared and the Fisher’s exact tests were performed. Continuous variables were compared with the Mann-Whitney U test or the Student’s t-test in case of normal distribution. Continuous variables are expressed as median and range throughout the text, unless stated otherwise. Graft and patient survival were compared by Log-rank test. Survival times, as primary endpoint of the study, are reported as estimated Kaplan-Meier median survival times, unless stated otherwise. *P*-values ≤0.050 were considered as statistically significant.

Figures were created with GraphPad Prism (version 9.1.0 for Windows, GraphPad Software, La Jolla, CA USA).

All methods were carried out in accordance with relevant guidelines and regulations.

## Results

### Recipients and donors

Seventeen males and ten females with a median age of 54 (35–65) years underwent HKTx. Except for the rate of prior dialysis (18 (66.7%) versus 27 (100%); *p* < 0.001) and, accordingly, the time on dialysis prior to transplant (9 (0–66) versus 86 (12–190) months; *p* < 0.001), recipient characteristics were not significantly different when compared to the patients undergoing solitary KTx (Table 1).

**Table 1:** Comparison of selected variables of patients undergoing either simultaneous heart-kidney or solitary kidney transplantation

Variables			HKTx			KTx			p-value
			Mean; Median (Range)	N (%)	M. v.	Mean; Median (Range)	N (%)	M. v.	
**Recipient data**	Age (in years)		52.52; 54 (35-65)		0 (0)	55.67; 58 (34-70)		0 (0)	0.222
	Male gender			17 (63.0)			18 (66.7)		0.776
	Height (in cm)		174.63; 173 (160-192)			171.63; 172 (156-184)			0.166
	Weight (in kg)		74.57; 72 (44-106)			73.01; 72.5 (44-103)			0.769
	BMI (in kg/m^2^)		24.29; 24.77 (15.59-31.44)			24.68; 24.92 (17.70-32.00)			0.711
	Cause of renal failure	Nephritis		8 (29.6)			11 (40.7)		n.a.
		Hypertension		3 (11.1)			1 (3.7)		
		CNI toxicity		3 (11.1)			0 (0)		
		Cystic kidney disease		3 (11.1)			1 (3.7)		
		Diabetes		3 (11.1)			3 (11.1)		
		Focal segmental glomerulosclerosis		1 (3.7)			2 (7.4)		
		Cardiorenal syndrom		3 (11.1)			0 (0)		
		Others		2 (7.4)			8 (29.6)		
		Undefined		1 (3.7)			1 (3.7)		
	Cause of cardiac failure	Dilated CM		15 (55.6)			n.a.		n.a.
		Ischemic CM		8 (29.6)					
		Others/Uncertain		4 (14.8)					
	Number of cardiovascular risk factors		1.26; 1 (0-5)			1.93; 2 (1-3)			**0.004**
	Previous HTx			3 (11.1)			0 (0)		0.105
	Previous KTx			1 (3.7)			2 (7.4)		0.549
	Previous cardiac operation requiring sternotomy			12 (44.4)			n.a.		n.a.
	Prior dialysis			18 (66.7)			27 (100)		**<0.001**
	Time on dialysis (in months)		20.22; 9 (0-66)			85.85; 86 (12-190)			**<0.001**
	GFR prior Tx for patients without prior dialysis (in ml/min)		22.67; 21 (15-35)			n.a.			n.a.
	PRA prior Tx (in %)		4.89; 0 (0-59)			2.04; 0 (0-55)			0.052
	Highest PRA (in %)		7.70; 0 (0-59)			5.00; 0 (0-88)			0.453
**Donor data**	Age (in years)		41.56; 47 (16-59)			43.41; 49 (18-62)			0.550
	Male gender			21 (77.8)			20 (74.1)		0.750
	Height (in cm)		175.85; 175 (165-190)			176.71; 176.5 (160-190)		3 (11.1)	0.688
	Weight (in kg)		80.22; 78 (60-120)			79.17; 75 (61-110)			0.740
	BMI (in kg/m^2^)		25.87; 24.84 (21.91-35.92)			25.34; 24.69 (20.88-32.65)			0.491
	Creatinine (in μmol/l)		72.12; 67.5 (35-181)		1 (3.7)	123.37; 80 (31-725)		0 (0)	0.071
	ICU ventilation (in days)		4.85; 3.5 (1-18)			4.85; 4 (1-15)			0.634
**Surgical details**	Number of HLA mismatches		4.15; 4 (2-6)		0 (0)	3.70; 4 (2-5)			0.159
	Operation time (in min)		116.78; 101.00 (60-225)			136.70; 134 (67-205)			**0.022**
	Cold ischemia time KTx (in min)		973.63; 900 (255-2084)			1125.74; 1118 (308-2286)			0.269
	PRBC (yes)			18 (66.7)	1 (3.7)		2 (7.4)		**<0.001**
	Number of PRBC		1.89; 2 (1-4)			1.50; 1.5 (1-2)			**<0.001**
	FFP (yes)			13 (48.1)			1 (3.7)		**<0.001**
	Number of FFP		3.23; 3 (1-9)			5.00; 5 (5-5)			**<0.001**

HKTx: simultaneous heart and kidney transplantation; KTx: kidney transplantation; BMI: body mass index CNI: calcineurin inhibitor; CM: cardiomyopathy; HTx: heart transplantation; GFR: glomerular filtration rate; PRA: panel reactive antibodies; ICU: intensive care unit; HLA: human leukocyte antigen; PRBC: packed red blood cells; FFP: fresh frozen plasma; M.v.: missing values; n.a.: not applicable/not applied. Bold values indicate statistical significance.

Donors of recipients undergoing HKTx showed lower creatinine concentrations at the time of organ recovery by trend (67.5 (35–181) μmol/l) versus 80 (31–725) μmol/l); *p* = 0.071). Further selected donor characteristics were not different between both groups (Table 1).

Of note, previous cardiac operations requiring sternotomy were carried out in twelve cases (including three previous cardiac transplants) prior HKTx. Additional Table 1 summarizes the type of cardiac operations performed prior to HKTx. Subgroup analyses did not reveal relevant differences with respect to specific recipient or donor characteristics (Table 2 and Additional Table 2).

**Table 2:** Comparison of variables of patients undergoing either simultaneous heart-kidney transplantation with prior cardiac surgery or solitary kidney transplantation

Variables			HKTx with prior cardiac surgery			KTx			p-value
			Mean; Median (Range)	N (%)	M. v.	Mean; Median (Range)	N (%)	M. v.	
**Recipient data**	Age (in years)		52.83; 55.5 (37-63)		0 (0)	55.67; 58 (34-70)		0 (0)	0.399
	Male gender			9 (75.0)			18 (66.7)		0.719
	Height (in cm)		178.17; 177.5 (172-192)			171.63; 172 (156-184)			**0.015**
	Weight (in kg)		80.58; 74.75 (60-106)			73.01; 72.5 (44-103)			0.127
	BMI (in kg/m^2^)		25.31; 24.42 (20.28-30.64)			24.68; 24.92 (17.70-32.00)			0.628
	Cause of renal failure	Nephritis		3 (25.0)			11 (40.7)		n.a.
		Hypertension		1 (8.3)			1 (3.7)		
		CNI toxicity		3 (25.0)			0 (0)		
		Cystic kidney disease		1 (8.3)			1 (3.7)		
		Diabetes		0 (0)			3 (11.1)		
		Focal segmental glomerulosclerosis		1 (8.3)			2 (7.4)		
		Cardiorenal syndrom		2 (16.7)			0 (0)		
		Others		0 (0)			8 (29.6)		
		Undefined		1 (8.3)			1 (3.7)		
	Cause of cardiac failure	Dilated CM		4 (33.3)			n.a.		n.a.
		Ischemic CM		5 (41.7)					
		Others/Uncertain		3 (25.0)					
	Number of cardiovascular risk factors		1.58; 1 (0-3)			1.93; 2 (1-3)			0.210
	Previous HTx			3 (25.0)			0 (0)		**0.024**
	Previous KTx			0 (0)			2 (7.4)		1.000
	Prior dialysis			5 (41.7)			27 (100)		**<0.001**
	Time on dialysis (in months)		11.23; 0 (0-57)			85.85; 86 (12-190)			**<0.001**
	GFR prior Tx for patients without prior dialysis (in ml/min)		20.57; 20 (15-27)			n.a.			n.a.
	PRA prior Tx (in %)		10.25; 0 (0-59)			2.04; 0 (0-55)			**0.014**
	Highest PRA (in %)		11.50; 0 (0-59)			5.00; 0 (0-88)			0.180
**Donor data**	Age (in years)		42.08; 45.5 (19-57)			43.41; 49 (18-62)			0.642
	Male gender			11 (91.7)			20 (74.1)		0.394
	Height (in cm)		179.00; 180.0 (170-190)			176.71; 176.5 (160-190)		3 (11.1)	0.377
	Weight (in kg)		83.33; 80.0 (65-120)			79.17; 75 (61-110)			0.398
	BMI (in kg/m^2^)		25.94; 24.76 (21.91-35.92)			25.34; 24.69 (20.88-32.65)			0.655
	Creatinine (in μmol/l)		79.17; 71 (43-181)			123.37; 80 (31-725)		0 (0)	0.425
	ICU ventilation (in days)		4.33; 2 (1-18)			4.85; 4 (1-15)			0.271
**Surgical details**	Number of HLA mismatches		4.00; 4 (3-6)			3.70; 4 (2-5)			0.447
	Operation time (in min)		136.70; 134 (67-205)			139.75; 134 (85-225)			0.820
	Cold ischemia time KTx (in min)		1161.50; 1224.5 (657-2084)			1125.74; 1118 (308-2286)			0.845
	PRBC (yes)			8 (66.7)			2 (7.4)		**<0.001**
	Number of PRBC		1.88; 2 (1-3)			1.50; 1.5 (1-2)			**0.002**
	FFP (yes)			7 (58.3)			1 (3.7)		**<0.001**
	Number of FFP		4.00; 4 (2-9)			5.00; 5 (5-5)			**0.008**

HKTx: simultaneous heart and kidney transplantation; KTx: kidney transplantation; BMI: body mass index CNI: calcineurin inhibitor; CM: cardiomyopathy; HTx: heart transplantation; GFR: glomerular filtration rate; PRA: panel reactive antibodies; ICU: intensive care unit; HLA: human leukocyte antigen; PRBC: packed red blood cells; FFP: fresh frozen plasma; M.v.: missing values; n.a.: not applicable/not applied. Bold values indicate statistical significance.

### Surgical procedure

Implantation times of renal grafts were shorter in patients undergoing HKTx (101 (60–225) minutes versus 134 (67–205) minutes; *p* = 0.022), as were cold ischemia times (900 (255–2084) minutes versus 1118 (308–2286) minutes; *p* = 0.269). The rate of transfusion of packed red blood cells and fresh frozen plasma as well as the number of units transfused were significantly elevated in HKTx recipients (*p* < 0.001). Table 1 gives an overview of selected surgical details for both groups. Of note, patients undergoing HKTx with prior cardiac surgery did not show additional significant differences regarding the surgical procedure, when compared to solitary KTx (Table 2), however; comparison with patients undergoing HKTx without prior cardiac surgery revealed prolonged implantation of the heart grafts (280.5 (190–584) minutes versus 216 (110–430) minutes; *p* = 0.007), the renal grafts (134 (85–225) minutes versus 100 (60–130) minutes; *p* = 0.004), and longer cold ischemia times of the latter (1224.5 (657–2084) minutes versus 805 (255–1545) minutes; *p* = 0.034). Furthermore, patients undergoing HKTx with prior cardiac surgery experienced severe hypotension (defined as mean arterial pressure < 55 mmHg) during and after cardiopulmonary bypass more frequently and more prolonged as patients without prior cardiac surgery (Additional Table 2).

### Immunosuppression

The immunosuppressive regimen of both groups changed over the time period analyzed. After HKTx, induction therapy consisted of anti-thymocyte globulin or basiliximab accompanied by steroids, calcineurin inhibitors (cyclosporine A or tacrolimus) and anti-proliferative agents, such as azathioprine or mycophenolate mofetil. Since 2004 nearly all patients received a standard immunosuppression of anti-thymocyte globulin, tacrolimus, mycophenolate mofetil and steroids. Only in one patient with prior HTx everolimus was continued instead of standard anti-proliferative agents.

With respect to patients undergoing solitary KTx, induction therapy with basiliximab was performed since 2002. Two patients received anti-thymocyte globulin and one patient alemtuzumab. Long-term therapy was based on cyclosporine A until 2017. Since then, tacrolimus was mainly used as calcineurin inhibitor. In 2005, triple therapy was established including mycophenolate mofetil, everolimus or mycophenolic acid in addition to steroids.

### Postoperative course and outcome

Median follow-up of patients was 100.33 (0.46–362.09) months. The time spent in ICU (5.5 (1–89) days versus 1 (0–8) days; *p* < 0.001) and in hospital (27 (14–236) days versus 20 (8–56); *p* = 0.022) were significantly prolonged in patients undergoing HKTx.

In the immediate postoperative course 4 of 27 patients undergoing solitary KTx experienced a total of five surgical revisions due to wound infection (*n* = 3), abnormal duplex sonography (*n* = 1) and insufficiency of the ureteral anastomosis (*n* = 1). Patients after simultaneous HKTx suffered from a higher frequency of postoperative complications. With regard to the kidney transplant, 10 of 27 patients underwent eleven surgical revisions, due to postoperative hematoma (*n* = 3), hemorrhage (*n* = 2), wound infection (*n* = 3) and abnormal duplex sonography (*n* = 3).

Furthermore, eight patients after HKTx underwent 15 thoracotomies for early complications, such as cardiac tamponade (*n* = 10), pericardial empyema (*n* = 1), postoperative hemorrhage (*n* = 1) and deep sternal wound infection (*n* = 3).

We observed a similar distribution of medical complications between both groups in the further course: Viral infections, such as cytomegalovirus, herpes zoster, BK virus and influenza, were observed in eight patients after KTx and six patients after HKTx. Bacterial infections (including severe urinary tract infections and pneumonia) were observed in eight patients after KTx and seven patients after HKTx. Five patients after KTx and two patients after HKTx developed post-transplant diabetes mellitus. Six patients after KTx and eight patients after HKTx suffered from different malignancies (predominantly affecting the skin, 2 to 12 years and 4 to 19 years after transplant, respectively).

Primary nonfunction was not observed in the included patients after solitary KTx. Four patients lost their grafts as a result of chronic graft failure/chronic vascular rejection (*n* = 3) and amyloidosis (*n* = 1). Nine grafts were lost due to death, with none of the patients dying within the primary hospital stay.

Five patients (18.5%) after HKTx experienced primary nonfunction with respect to the renal grafts (with only one patient still alive at the time of follow-up). Of note, four of these five patients were high-risk patients with prior cardiac surgery requiring sternotomy. Accordingly, the rate of subsequent dialysis in the postoperative course was significantly higher after HKTx (13 (48.1%) versus 6 (22.2%); *p* = 0.044). Two further patients lost their grafts due to polyoma nephropathy. One patient lost renal graft function after successful resuscitation and died approximately two months later due to sepsis. Ten patients after HKTx suffered from deaths with functioning graft (DWFG). Of these, one died within the primary hospital stay due to sepsis.

The causes of death for patients of both groups in the further follow-up were mainly related to cardiovascular complications, but are difficult to assign retrospectively for a large proportion of the included patients due to multiple underlying comorbidities and partially poor workup at the time of death.

**Fig. 1:**
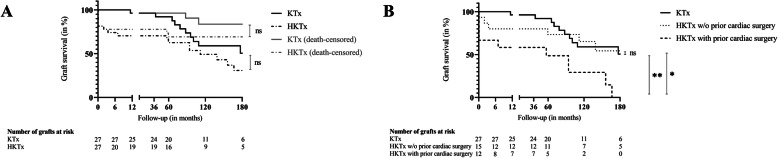
Kidney graft survival after solitary kidney or simultaneous heart-kidney transplantation. Kidney graft survival after solitary kidney transplantation (KTx) or simultaneous heart-kidney transplantation (HKTx) (non-censored and censored for death) (**A**) and broken down into subgroups with regard to prior cardiac surgery (non-censored for death) (**B**). A comparison of survival curves shows the rapid decline in kidney graft survival, mainly due to the high rate of primary nonfunction and in-hospital mortality in patients undergoing HKTx. The further course depicts an additional decline in graft survival in patients undergoing HKTx with prior cardiac surgery requiring sternotomy, when compared to solitary KTx or HKTx with or without (w/o) prior cardiac surgery. ns not significant. * HKTx w/o prior cardiac surgery versus HKTx with prior cardiac surgery (*p* = 0.016). ** KTx versus HKTx with prior cardiac surgery (*p* = 0.001)

Despite lower five-year kidney graft survival (62.6% versus 92.1%; 111.73 versus 183.08 months; *p* = 0.189; Fig. 1A), graft function over time (monitored by eGFR; Table 3), and patient survival (138.90  versus 192.71 months; *p* = 0.128) were not significantly inferior after HKTx in general.

**Table 3:** Comparison of graft and patient outcome after either simultaneous heart-kidney or solitary kidney transplantation

Variables		HKTx			KTx			p-value
		Mean; Median (Range)	N (%)	M. v.	Mean; Median (Range)	N (%)	M. v.	
**Outcome**	1-month eGFR (in ml/min)	61.20; 53.43 (25.13-118.89)		11 (40.7)	44.23; 42.07 (10.23-92.15)		1 (3.7)	0.066
	3-month eGFR (in ml/min)	49.98; 51.64 (23.07-72.98)		9 (33.3)	46.60; 44.83 (19.44-97.48)		2 (7.4)	0.584
	6-month eGFR (in ml/min)	45.87; 42.27 (18.81-91.41)		7 (25.9)	49.38; 42.22 (16.38-105.52)		2 (7.4)	0.571
	1-year eGFR (in ml/min)	46.19; 45.01 (13.60-82.95)		7 (25.9)	51.53; 49.09 (21.25-80.24)		3 (11.1)	0.358
	2-year eGFR (in ml/min)	44.58; 42.15 (14.75-79.89)		8 (29.6)	49.44; 47.22 (22.13-77.70)		4 (14.8)	0.360
	3-year eGFR (in ml/min)	44.24; 36.99 (12.48-85.31)		8 (29.6)	47.34; 47.79 (27.29-78.49)		3 (11.1)	0.584
	5-year eGFR (in ml/min)	43.59; 37.73 (19.92-93.55)		12 (44.4)	47.82; 43.15 (21.28-90.58)		5 (18.5)	0.421
	10-year eGFR (in ml/min)	46.11; 48.59 (15.98-77.03)		18 (66.7)	49.18; 41.46 (30.13-88.25)		16 (59.3)	0.732
	Subsequent dialysis		13 (48.1)	0 (0)		6 (22.2)	0 (0)	**0.044**
	ICU stay (in days)	15.62; 5.5 (1-89)		1 (3.7)	2.07; 1 (0-8)			**<0.001**
	Hospital stay (in days)	56.78; 27 (14-236)		0 (0)	21.59; 20 (8-56)			**0.022**
	Surgical complications		13 (48.1)			4 (14.8)		**0.008**
	Primary nonfunction (renal graft)		5 (18.5)			0 (0)		0.051
	In-hospital mortality		6 (22.2)			0 (0)		**0.023**

HKTx: simultaneous heart and kidney transplantation; KTx: kidney transplantation; HTx: heart transplantation; eGFR: estimated glomerular filtration rate; ICU: intensive care unit; M.v.: missing values. Bold values indicate statistical significance.

However, HKTx with prior cardiac surgery resulted in poor graft and patient survival (57.00 and 94.09 months, respectively) when compared to patients undergoing solitary KTx (183.08 and 192.71 months; *p* < 0.001; Fig. 1B) and simultaneous HKTx without prior surgery (203.22 and 203.22 months; *p* = 0.016 and 0.019, respectively; Fig. 1B; Additional Table 3) most probably explained by the high rate of primary nonfunction (4 of 12 patients; 33.0% versus 0 of 27 patients; 0% after KTx; *p* = 0.006) and in-hospital mortality (3 of 12 patients; 25.0% versus 0 of 27 patients; 0%; *p* = 0.024) (see also Table 4).

**Table 4:** Comparison of graft and patient outcome after either simultaneous heart-kidney with prior cardiac surgery or solitary kidney transplantation

Variables		HKTx with prior cardiac surgery			KTx			p-value
		Mean; Median (Range)	N (%)	M. v.	Mean; Median (Range)	N (%)	M. v.	
**Outcome**	1-month eGFR (in ml/min)	67.03; 69,47 (37.34-93.19)		6 (50.0)	44.23; 42.07 (10.23-92.15)		1 (3.7)	**0.019**
	3-month eGFR (in ml/min)	51.16; 52.30 (23.07-72.63)		5 (41.7)	46.60; 44.83 (19.44-97.48)		2 (7.4)	0.622
	6-month eGFR (in ml/min)	49.32; 41.81 (18.81-91.41)		4 (33.3)	49.38; 42.22 (16.38-105.52)		2 (7.4)	0.995
	1-year eGFR (in ml/min)	49.18; 47.72 (13.6-79.08)		4 (33.3)	51.53; 49.09 (21.25-80.24)		3 (11.1)	0.773
	2-year eGFR (in ml/min)	48.27; 46.18 (24.84-69.80)		5 (41.7)	49.44; 47.22 (22.13-77.70)		4 (14.8)	0.877
	3-year eGFR (in ml/min)	46.84; 36.16 (13.81-85.31)		5 (41.7)	47.34; 47.79 (27.29-78.49)		3 (11.1)	0.951
	5-year eGFR (in ml/min)	43.10; 30.99 (19.92-73.83)		7 (58.3)	47.82; 43.15 (21.28-90.58)		5 (18.5)	0.620
	10-year eGFR (in ml/min)	45.88; 53.81 (15.98-67.86)		9 (75.0)	49.18; 41.46 (30.13-88.25)		16 (59.3)	0.817
	Subsequent dialysis		6 (50.0)	0 (0)		6 (22.2)	0 (0)	0.133
	ICU stay (in days)	11.33; 8.5 (1-36)			2.07; 1 (0-8)			**<0.001**
	Hospital stay (in days)	62.50; 28 (14-236)			21.59; 20 (8-56)			**0.012**
	Surgical complications		7 (58.3)			4 (14.8)		**0.017**
	Primary nonfunction (renal graft)		4 (33.3)			0 (0)		**0.006**
	In-hospital mortality		3 (25.0)			0 (0)		**0.024**

HKTx: simultaneous heart and kidney transplantation; KTx: kidney transplantation; HTx: heart transplantation; eGFR: estimated glomerular filtration rate; ICU: intensive care unit; M.v.: missing values. Bold values indicate statistical significance.

Of note, patient survival after simultaneous HKTx was superior by trend when compared to a group of 27 patients undergoing solitary HTx, matched for era of transplantation, prior cardiac surgery, cause of cardiac failure and donor and recipient specific biometrics (138.90 versus 91.36 months; *p* = 0.509; Additional Fig. 1).

## Discussion

Increasing numbers of simultaneous HKTx in the recent past demonstrate the establishment of this procedure in patients with end-stage heart disease and accompanying renal failure. In the absence of universally accepted guidelines for eligibility for HKTx, indications and outcomes have to be reviewed critically to ensure optimal utilization of scarce resources in times of ongoing organ shortage crisis.

In our study we therefore investigated the outcome of patients undergoing HKTx in comparison to patients undergoing solitary KTx, focusing on renal graft performance and survival. Previous investigations on the matter demonstrated that graft damage due to the necessity of complex and prolonged ICU treatment as well as early renal graft loss due to increased in-hospital mortality following HKTx are major limitations for the success of this combined approach [1, 5, 6, 14]. Significantly lengthened ICU treatment, frequent necessity of subsequent dialysis and a considerable rate of primary nonfunction (18.5%; 5 of 27 patients) and in-hospital mortality (22%, 6 of 27 patients) in our patients undergoing HKTx corroborate these previous findings. Of note, implantation times of renal grafts were comparatively short [15, 16] in both groups most likely owed to the experience of a high-volume center with more than 140 KTx annually.

Perhaps unsurprisingly, HKTx was accompanied by an increased rate of intraoperative transfusions as a result of prior implantation of the cardiac graft, which has been shown to trigger complement activation and inflammation by hem release and increase of plasma-free hemoglobin in the past [17].

Similarly detrimental effects on renal function have been reported from the use of ventricular assist devices, venous-arterial extracorporeal membrane oxygenation support and veno-venous hemofiltration all frequently part of the treatment of hemodynamic instable patients after cardiac transplantation [18, 19].

To avoid hemodynamic instability and negative influence of its treatment during kidney implantation and the early post-transplant period or even graft loss due to early mortality some authors suggest a staged procedure [5, 20, 21], implying that the renal engraftment is delayed for several hours. However, a staged procedure increases ischemic allograft time and is therefore associated with impaired renal function and reduced long-term graft survival [22–24] and thus others recommend implantation immediately following thoracic closure [25].

Our preferred modus operandi is to decide on the beginning of renal transplantation for each case individually: Patients undergoing short and complication-free cardiac transplant (i.e. short bypass time and sparse transfusion of blood products) benefit from short ischemic time of the renal graft by starting implantation immediately upon cardiac engraftment. For other cases, we prefer a staged strategy to allow hemodynamic stabilization and correction of coagulation abnormalities.

Previous studies of patients receiving HKTx reported considerably impaired renal graft and patient survival after the first year of transplantation. After five years, patient survival was reported between 53 to 80% versus 84 to 86% in case of solitary KTx, whereas differences in renal graft survival were slightly less pronounced (69% versus 76%) [1, 4–7]. Our study demonstrates similar results after one and five years, however; significant differences in graft and patient survival diminished over time, which is mainly explained by a lower rate of DWFG following the first year after HKTx. DWFG is common after KTx and responsible for more than one third of all graft losses, also contributing to the lack of improvement of long-term results in recent years [26], and is mainly caused by cardiovascular disease [26] providing reasonable explanation for a decrease of DWFG after successful HTx and HKTx, respectively.

Apart from mere survival, we observed similar graft function after one, five and ten years after HKTx when compared to solitary KTx.

As our study demonstrates favorable results after HKTx in comparison with solitary KTx, except for an early postoperative discrepancy regarding graft and patient survival, we aimed to elucidate potential patients at risk for poor outcomes. Subgroup analyses of the patients undergoing HKTx revealed prior cardiac surgery requiring sternotomy as a significant factor contributing to primary nonfunction, early graft loss and recipient death, respectively. In addition, survival over the further course in these patients was also inferior when compared to patients undergoing HKTx without prior cardiac surgery or solitary KTx. An explanation for this observation could be the significantly longer CIT and operation time, respectively, as well as increased hemodynamic instability (reflected by a trend towards increased transfusion of blood products as well as a higher incidence and longer durations of severe hypotension during and after HTx). Although the significance of our results is limited by the retrospective nature of the study and the relatively small number of included cases, a simultaneous approach of HKTx for these patients might not be an optimal solution and alternative concepts have to be considered.

In any case, predicting further development of an impaired renal function after HTx is extremely difficult prior engraftment, and thus requires critical appraisal [27–29]. Obviously, poor renal function was frequently reported as risk factor for post-transplant dialysis dependent acute renal failure, however; further intra- and perioperative factors such as high-dose vasopressor therapy or mechanical circulatory support, re-operation due to hemorrhage, intubation time of more than 24 hours, use of ventricular assistant device or previous cardiac surgery demonstrated adverse impact on posttransplant kidney function after HTx [27–29]. Moreover, of those recipients with normal or impaired renal function (not meeting the requirements for KTx listing) before solitary HTx, that develop early postoperative kidney dysfunction, up to 88% experience recovery of renal function and no association with later development of end-stage renal failure was detected [27, 29].

In summary, when performing simultaneous HKTx two aspects have to be considered with respect to adequate utilization of renal grafts: 1.) Risk of severe perioperative graft damage inflicted by prolonged hemodynamic instability and early graft loss as a result of patient death. 2.) Dispensable renal engraftment, because of potential renal recovery.

As a solution to these issues, a “kidney-after-heart” program for patients suffering from severe renal insufficiency at the time of listing for HTx has been suggested. According to our data, this would be especially useful in recipients with increased perioperative risk (such as prior cardiac surgery requiring sternotomy). After successful HTx and consistent severe renal failure, thus excluding those patients with recovering renal function, the program should offer the possibility for an additional KTx six to twelve months after HTx - comparable to the already established “kidney-after-liver” program at Eurotransplant.

Needless to say, immunological advantages of a same donor organ transplantation [30] would be lost in such a concept, however, previous investigations demonstrated encouraging results on kidney after heart transplantation [31, 32], with the most recent study originating from the United Network for Organ Sharing database data showing even superior patient survival in comparison to HKTx [33].

In other words, HKTx should be restricted to patients without any prior cardiac surgery (or other factors indicating high-risk HTx) suffering from advanced renal failure without the prospect of improvement, reflected by a multiannual history of intrinsic renal disease having resulted in pre-transplant renal replacement therapy or alternatively biopsy-based evidence for advanced structural renal damage in combination with a GFR of lower than 30 ml/min.

## Conclusions

Our data demonstrates the increased rate of early kidney graft loss and thus significantly inferior graft survival in high-risk patients undergoing HKTx.

Thus, we advocate for a “kidney-after-heart” program in such patients to ensure responsible and reasonable utilization of scarce resources in times of ongoing organ shortage crisis.

## Supplementary Information

**Additional file 1 Fig. 1.** Patient survival after solitary heart transplantation (HTx) or simultaneous heart-kidney transplantation (HKTx). Patients from both groups were matched for era of transplantation, prior cardiac surgery, cause of cardiac failure and donor and recipient specific biometrics. Of note, patients undergoing HTx did not suffer from end-stage renal failure at the time of transplantation. Despite a trend towards a superior patient survival after five years in patients undergoing HKTx, both groups showed comparable long-term outcome (*p* = 0.509). ns not significant.

**Additional file 2 Table 1.** Overview of cardiac operations requiring sternotomy performed prior to HKTx.

**Additional file 3 Table 2.** Comparison of variables of patients undergoing simultaneous heart-kidney transplantation either with or without prior cardiac surgery (Page 1 and 2).

**Additional file 4 Table 3.** Comparison of graft and patient outcome after simultaneous heart-kidney transplantation with or without prior cardiac surgery.

## Data Availability

Due to the limited number of patients included into the study and the specific setting – especially in case of HKTx – the datasets are not shared publicly to ensure individual privacy. However, the datasets used and/or analyzed during the current study are available from the corresponding author on reasonable request.

## Abbreviations

HKTx: simultaneous heart and kidney transplantation
KTx: kidney transplantation
HTx: heart transplantation
eGFR: estimated glomerular filtration rate
ICU: intensive care unit
DWFG: deaths with functioning graft
