# Supplementary figures and images for: Simultaneous heart-kidney transplantation results in respectable long-term outcome but a high rate of early kidney graft loss in high-risk recipients – a European single center analysis
Source: BMC Nephrol. 2021 Jul 9;22:258. doi: 10.1186/s12882-021-02430-x (PMC8268408; doi:10.1186/s12882-021-02430-x)

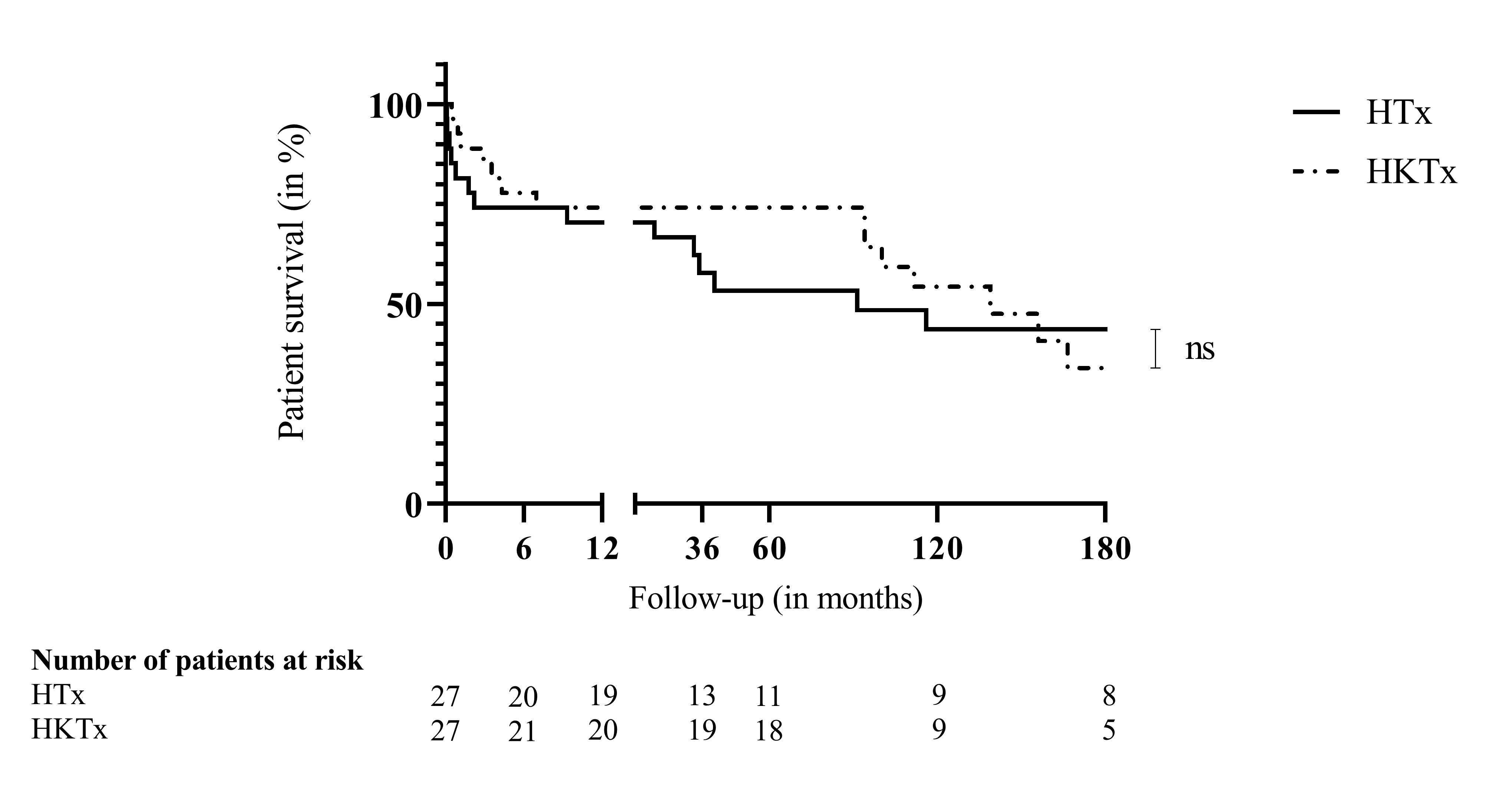

Supplement: Supplementary file 1 — Additional file 1 Fig. 1. Patient survival after solitary heart transplantation (HTx) or simultaneous heart-kidney transplantation (HKTx). Patients from both groups were matched for era of transplantation, prior cardiac surgery, cause of cardiac failure and donor and recipient specific biometrics. Of note, patients undergoing HTx did not suffer from end-stage renal failure at the time of transplantation. Despite a trend towards a superior patient survival after five years in patients undergoing HKTx, both groups showed comparable long-term outcome (p = 0.509). ns not significant. [file 12882_2021_2430_MOESM1_ESM.tif]
