# Supplementary material for: Simultaneous heart-kidney transplantation results in respectable long-term outcome but a high rate of early kidney graft loss in high-risk recipients – a European single center analysis
Source: BMC Nephrol. 2021 Jul 9;22:258. doi: 10.1186/s12882-021-02430-x (PMC8268408; doi:10.1186/s12882-021-02430-x)
Supplement: Supplementary file 2 — Additional file 2 Table 1. Overview of cardiac operations requiring sternotomy performed prior to HKTx. [file 12882_2021_2430_MOESM2_ESM.pdf]

Additional Table 1. Overview of cardiac operations requiring sternotomy performed prior to HKTx

Pat. No.	Previous cardiac operation	Time upon last sternotomy (in months)
1	Heart transplant (1986), Tricuspid valve replacement (1994)	19
2	Coronary artery bypass graft (n=4; 1995)	15
3	Coronary artery bypass graft (1988, 1994)	54
4	Coronary artery bypass graft (1998)	58
5	Heart transplant (1998); Replacement of ascending aorta and aortic valve reconstruction (1998), aortic valve replacement (2003)	24
6	Implantation of a cardiac assist device (Heartmate I; 2005)	15
7	Heart transplant (1994)	152
8	Cardiomyoplasty (Latissimus dorsi muscle flap; 1998)	138
9	Coronary artery bypass graft (n=2; 2014), Implantation of a cardiac assist device (Heartmate II; 2014)	24
10	Aortic valve replacement (1992), Aortic and mitral valve replacement (2001)	204
11	Surgery for double outlet right ventricle (1982)	441
12	Mitral and tricuspid valve replacement (2019), Implantation of a biventricular cardiac assist device (total artificial heart, Heartmate III) after ventricle resection (2019)	9
