# Supplementary material for: Simultaneous heart-kidney transplantation results in respectable long-term outcome but a high rate of early kidney graft loss in high-risk recipients – a European single center analysis
Source: BMC Nephrol. 2021 Jul 9;22:258. doi: 10.1186/s12882-021-02430-x (PMC8268408; doi:10.1186/s12882-021-02430-x)
Supplement: Supplementary file 3 — Additional file 3 Table 2. Comparison of variables of patients undergoing simultaneous heart-kidney transplantation either with or without prior cardiac surgery (Page 1 and 2). [file 12882_2021_2430_MOESM3_ESM.pdf]

Additional Table 2. Comparison of variables of patients undergoing simultaneous heart-kidney transplantation either with or without prior cardiac surgery (Page 1 and 2)

Variables			HKTx with prior cardiac surgery			HKTx without prior cardiac operation			p-value
			**Mean; Median (Range)**	**N (%)**	**M. v.**	**Mean; Median (Range)**	**N (%)**	**M. v.**	
**Recipient data**	Age (in years)		52.83; 55.5 (37-63)		0 (0)	52.27; 54 (3-65)		0 (0)	0.873
	Male gender			9 (75.0)			8 (53.3)		0.424
	Height (in cm)		178.17; 177.5 (172-192)			171.80; 172 (160-189)			**0.036**
	Weight (in kg)		80.58; 74.75 (60-106)			69.76; 69 (44-96)			0.074
	BMI (in kg/m^2)		25.31; 24.42 (20.28-30.64)			23.47; 24.77 (15.59-31.44)			0.242
	Cause of renal failure	Nephritis		3 (25.0)			5 (33.3)		n.a.
		Hypertension		1 (8.3)			2 (13.3)		
		CNI toxicity		3 (25.0)			0 (0)		
		Cystic kidney disease		1 (8.3)			2 (13.3)		
		Diabetes		0 (0)			3 (20.0)		
		Focal segmental glomerulosclerosis		1 (8.3)			0 (0)		
		Cardiorenal syndrom		2 (16.7)			1 (6.7)		
		Others		0 (0)			2 (13.3)		
		Undefined		1 (8.3)			0 (0)		
	Cause of cardiac failure	Dilated CM		4 (33.3)			11 (73.3)		n.a.
		Ischemic CM		5 (41.7)			3 (20.0)		
		Others/Uncertain		3 (25.0)			1 (6.7)		
	Number of cardiovascular risk factors		1.58; 1 (0-3)			1.00; 1 (0-5)			0.065
	Previous HTx			3 (25.0)			0 (0)		0.075
	Previous KTx			0 (0)			1 (6.7)		1.000
	Prior dialysis			5 (41.7)			13 (86.7)		**0.037**
	Time on dialysis (in months)		11.23; 0 (0-57)			27.40; 29 (0-66)			**0.032**
	GFR prior Tx for patients without prior dialysis (in ml/min)		20.57; 20 (15-27)			30.00; 30 (25-35)			**0.032**
	PRA prior Tx (in %)		10.25; 0 (0-59)			0.60; 0 (0-6)			0.140
	Highest PRA (in %)		11.50; 0 (0-59)			4.67; 0 (0-53)			0.282
**Donor data**	Age (in years)		42.08; 45.5 (19-57)			41.13; 48.0 (16-59)			0.943
	Male gender			11 (91.7)			10 (66.7)		0.182
	Height (in cm)		179.00; 180 (170-190)			173.33; 172 (165-190)			**0.044**
	Weight (in kg)		83.33; 80.0 (65-120)			77.73; 75.0 (60-110)			0.323
	BMI (in kg/m^2)		25.94; 24.76 (21.91-35.92)			25.80; 25.71 (21.91-31.07)			0.792
	Creatinine (in µmol/l)		79.17; 71 (43-181)			66.07; 59 (35-124)			0.145
	ICU ventilation (in days)		4.33; 2 (1-18)			5.29; 4 (1-15)		1 (6.7)	0.297

Variables		HKTx with prior cardiac surgery			HKTx without prior cardiac operation			p-value
		Mean; Median (Range)	N (%)	M. v.	Mean; Median (Range)	N (%)	M. v.	
**Surgical details**	Number of HLA mismatches	4.00; 4 (3-6)			4.27; 4 (2-6)		0 (0)	0.545
	Operation time HTx (in min)	327.00; 280.5 (190-584)			231.13; 216 (110-430)			**0.007**
	Operation time KTx (in min)	139.75; 134 (85-225)			98.40;100 (60-130)			**0.004**
	Bypass time (in min)	175.25; 164 (96-280)			130.87; 127 (61-201)			**0.042**
	Time between HTx and KTx reperfusion (in min)	902.50; 810.5 (482-1799)			648.07; 644 (87-1336)		1 (6.7)	0.094
	Cold ischemia time HTx (in min)	259.00; 199 (175-476)			193.67; 201 (127-260)		0 (0)	0.256
	Cold ischemia time KTx (in min)	1161.50; 1224.5 (657-2084)			823.33; 805 (255-1545)			**0.034**
	PRBC KTx (yes)		8 (66.7)			10 (66.7)	1 (6.7)	1.000
	Number of PRBC KTx	1.88; 2 (1-3)			1.90; 2 (1-4)			0.940
	FFP KTx (yes)		7 (58.3)			6 (40.0)		0.431
	Number of FFP KTx	4.00; 4 (2-9)			2.33; 1.5 (1-6)			0.212
	PRBC HTx (yes)		10 (83.3)			11 (73.3)	0 (0)	0.662
	Number of PRBC HTx	7.60; 6.5 (3-13)			5.64; 5 (2-17)			0.139
	FFP HTx (yes)		12 (100)			14 (93.3)		1.000
	Number of FFP HTx	7.83; 4 (2-20)			4.21; 4 (1-16)			0.152
	Vasopressors during CPB (yes)		9 (75.0)	1 (8.3)		10 (66.7)	1 (6.7)	0.661
	Vasopressors after CPB (yes)		10 (83.3)	1 (8.3)		9 (60.0)	3 (20.0)	0.590
	Vasopressors during KTx (yes)		9 (75.0)	0 (0)		6 (40.0)	3 (20.0)	0.400
	Severe hypotension during CPB (MAP < 55 mmHg; yes)		9 (75.0)			6 (40.0)	1 (6.7)	0.098
	Severe hypotension during CPB (MAP < 55 mmHg; in min)	48.67; 11 (0-271)			11.00; 0 (0-44)			0.156
	Severe hypotension after CPB (MAP < 55 mmHg; yes)		5 (41.7)			0 (0)		**0.012**
	Severe hypotension after CPB (MAP < 55 mmHg; in min)	16.83; 0 (0-121)			0; 0 (0-0)			**0.009**
	Severe hypotension during KTx (MAP < 55 mmHg; yes)		2 (16.7)			2 (13.3)	2 (13.3)	1.000
	Severe hypotension during KTx (MAP < 55 mmHg; in min)	2.75; 0 (0-27)			1.31; 0 (0-12)			0.865

HKTx: simultaneous heart and kidney transplantation; KTx: kidney transplantation; BMI: body mass index CNI: calcineurin inhibitor; CM: cardiomyopathy; HTx: heart transplantation; GFR: glomerular filtration rate; PRA: panel reactive antibodies; ICU: intensive care unit; HLA: human leukocyte antigen; PRBC: packed red blood cells; FFP: fresh frozen plasma; CPB: cardiopulmonary bypass; MAP: mean arterial pressure; M.v.: missing values; n.a.: not applicable/not applied. Bold values indicate statistical significance.
