# Supplementary material for: Simultaneous heart-kidney transplantation results in respectable long-term outcome but a high rate of early kidney graft loss in high-risk recipients – a European single center analysis
Source: BMC Nephrol. 2021 Jul 9;22:258. doi: 10.1186/s12882-021-02430-x (PMC8268408; doi:10.1186/s12882-021-02430-x)
Supplement: Supplementary file 4 — Additional file 4 Table 3. Comparison of graft and patient outcome after simultaneous heart-kidney transplantation with or without prior cardiac surgery. [file 12882_2021_2430_MOESM4_ESM.pdf]

Additional Table 3. Comparison of graft and patient outcome after simultaneous heart-kidney transplantation with or without prior cardiac surgery

Variables		HKTx with prior cardiac surgery			HKTx without prior cardiac operation			p-value
		**Mean; Median (Range)**	**N (%)**	**M. v.**	**Mean; Median (Range)**	**N (%)**	**M. v.**	
**Outcome**	1-month eGFR (in ml/min)	67.03; 69,47 (37.34-93.19)		6 (50.0)	57.71; 47.69 (25.13-118.89)		5 (33.3)	0.548
	3-month eGFR (in ml/min)	51.16; 52.30 (23.07-72.63)		5 (41.7)	49.23; 46.90 (26.08-72.98)		4 (26.7)	0.817
	6-month eGFR (in ml/min)	49.32; 41.81 (18.81-91.41)		4 (33.3)	43.57; 42.34 (19.08-62.54)		3 (20.0)	0.483
	1-year eGFR (in ml/min)	49.18; 47.72 (13.6-79.08)		4 (33.3)	44.19; 41.55 (19.47-82.95)		3 (20.0)	0.582
	2-year eGFR (in ml/min)	48.27; 46.18 (24.84-69.80)		5 (41.7)	42.43; 40.69 (14.75-79.89)		3 (20.0)	0.479
	3-year eGFR (in ml/min)	46.84; 36.16 (13.81-85.31)		5 (41.7)	42.72; 37.09 (12.48-84.02)		3 (20.0)	0.699
	5-year eGFR (in ml/min)	43.10; 30.99 (19.92-73.83)		7 (58.3)	42.84; 39.69 (28.56-93.55)		5 (33.3)	0.594
	10-year eGFR (in ml/min)	45.88; 53.81 (15.98-67.86)		9 (75.0)	46.22; 43.04 (29.05-77.03)		9 (60.0)	0.982
	Subsequent dialysis		6 (50.0)			7 (46.7)	0 (0)	0.863
	ICU stay (in days)	11.33; 8.5 (1-36)			19.29; 5.5 (1-89)		1 (6.7)	0.899
	Hospital stay (in days)	62.50; 28 (14-236)			52.20; 20 (15-179)			0.347
	Surgical complications		7 (58.3)			6 (40.0)		0.343
	Primary nonfunction (renal graft)		4 (33.3)			1 (6.7)		0.139
	In-hospital mortality		3 (25.0)			3 (20.0)		1.000
	Graft survival HTx (months)	82.04; 94.02 (n.a.)			213.21; 203.22 (n.a.)			**0.017**
	HTx graft loss		9 (75.0)	0 (0)		7 (46.7)	0 (0)	0.239
	HTx graft rejection		2 (16.7)			4 (26.7)		0.662
	Graft survival KTx (months)	71.45; 57.00 (n.a.)			199.12; 203.22 (n.a.)			**0.016**
	KTx graft loss		10 (83.3)			8 (53.3)		0.217
	KTx graft rejection		1 (8.3)			1 (6.7)		1.000
	Patient survival (months)	86.59; 94.09 (n.a.)			213.21; 203.22 (n.a.)			**0.019**
	Death		9 (75.0)			7 (46.7)		0.239

HKTx: simultaneous heart and kidney transplantation; KTx: kidney transplantation; HTx: heart transplantation; eGFR: estimated glomerular filtration rate; ICU: intensive care unit; M.v.: missing values. Bold values indicate statistical significance.
